# New Insights into the Exploitation of *Vitis vinifera* L. cv. Aglianico Leaf Extracts for Nutraceutical Purposes

**DOI:** 10.3390/antiox9080708

**Published:** 2020-08-04

**Authors:** Fabiana Labanca, Immacolata Faraone, Maria Rosaria Nolè, Ruth Hornedo-Ortega, Daniela Russo, Maria Carmen García-Parrilla, Lucia Chiummiento, Maria Grazia Bonomo, Luigi Milella

**Affiliations:** 1 Dipartimento di Scienze, Università della Basilicata, v.le Ateneo Lucano 10, 85100 Potenza, Italy; fabiana.labanca@unibas.it (F.L.); immafaraone88@gmail.com (I.F.); maryr91@alice.it (M.R.N.); lucia.chiummiento@unibas.it (L.C.); mariagrazia.bonomo@unibas.it (M.G.B.); 2 Spinoff BioActiPlant s.r.l., Università della Basilicata, v.le Ateneo Lucano 10, 85100 Potenza, Italy; 3 Área de Nutrición y Bromatología, Facultad de Farmacia, Universidad de Sevilla, C/P., Garcıa Gonzalez 2, 41012 Sevilla, Spain; rhornedo@us.es (R.H.-O.); mcparrilla@us.es (M.C.G.-P.)

**Keywords:** Alzheimer’s disease, *Vitis vinifera*, antioxidant properties, enzymatic inhibitory activity, acetylcholinesterase, butyrylcholinesterase, tyrosinase, polyphenol compounds

## Abstract

The leaves of *Vitis vinifera* L. have been used for a long time in traditional medicine for the treatment of many ailments. Grape polyphenols, indeed, have been demonstrated to be able to defend against oxidative stress, responsible for various disorders such as cancer, diabetes and neurodegenerative diseases. The effects of different extraction techniques, Soxhlet (SOX), Accelerated Solvent (ASE 40, ASE 50) and Ultrasound Assisted Extraction (UAE) were studied in this work to evaluate their impact on the chemical profile and bioactive potential of *Vitis vinifera* L. (cv. Aglianico) leaf extracts. The phytochemical profile was investigated by HPLC-DAD and 9 phenolic compounds were identified and quantified in the extract. Moreover, the antioxidant, anticholinesterase and antityrosinase activities were evaluated. In detail, the total polyphenol content and antioxidant activity (2,2-diphenyl-1-picrylhydrazyl, Oxygen Radical Absorbance Capacities and *β*-Carotene Bleaching assays) were evaluated and compared to assess the Relative Antioxidant Capacity Index (RACI). To test the inhibitory activity of extracts towards cholinesterases, acetylcholinesterase (AChE) and butyrylcholinesterase (BChE) inhibition assays were performed. SOX and ASE 50 have shown the highest value of RACI, 0.76 and 0.65, respectively. Regarding enzymatic inhibitory activity, ASE 50 (IC_50_ = 107.16 ± 8.12 μg/mL) and SOX (IC_50_ = 171.34 ± 12.12 μg/mL) extracts exhibited the highest AChE and BChE inhibitory activity, respectively, while UAE (IC_50_ = 293.2 ± 25.6 μg/mL, followed by SOX (IC_50_ = 302.5 ± 38.3 μg/mL) showed the highest tyrosinase inhibition value. Our results demonstrated for the first time that Aglianico leaves are important sources of phenols that could be used to prevent oxidative stress and be potentially helpful in diseases treatable with tyrosinase and cholinesterase inhibitors, like myasthenia gravis or Alzheimer’s.

## 1. Introduction

Grapevine is one of the most widely cultivated plants in the world, with a global annual production of nearly 80 million tons in 2018 [1], destined for different purposes (wine, juice, table grapes, etc.). Grapevine can be considered an important source of bioactive compounds, mainly polyphenols [2]. *Vitis vinifera* L. leaves, commonly consumed as food in the Mediterranean area, are also used as a demulcent, cathartic, stomachic and diuretic. Moreover, it has been demonstrated to be useful in bilious dyspepsia, hemorrhage, dysuria, in chronic bronchitis, heart diseases and gout, while in folk medicine it prevents constipation. The extraction procedure is an important step in recovering bioactive compounds from plant matrices. Conventional (maceration, Soxhlet extraction) and non-conventional (ultrasound-assisted, microwave-assisted, accelerated solvent and subcritical water extraction techniques) approaches can be applied [3]. Extraction method effectiveness is evident when different methods are practiced on the same plant matrices by using the same solvent and extraction efficiency shows significant variations [4]. The extraction methods should be optimized not only for its total phytochemical profile but also for its biological effects. Several studies reported that the phenol composition of grapes is strongly related to geographical and climate factors as well as to grape variety [5]. *Vitis vinifera* L. cv. Aglianico is a variety of red grape cultivar cultivated in Southern Italy as Basilicata and Campania. In Basilicata the cultivation of Aglianico vineyards is mainly localized in the Vulture area and the wine awarded the Controlled Designation of Origin (CDO) label, in compliance with the legislation (EU 1971) [6]. Previous studies on Aglianico cultivar have largely investigated the phenolic composition of grape berries, berries skin and wine showing the presence of flavonoid (anthocyanins, procyanidins, flavonols, flavan-3-ols, flavones, flavonones) and non-flavonoid compounds (phenolic acids, stilbenes) [7,8,9,10], with antioxidant, antimicrobial, antiviral, anti-inflammatory properties [11,12]. Gabriele et al. [13] evaluated the effect of low sulphur dioxide concentrations on the chromatic properties, phytochemical composition and antioxidant activity of Aglianico red wines.

Nowadays, one of the biggest challenges in agricultural practices is to develop alternatives and valorize the huge amount of by-products. It is for this reason that the European Commission has established a directive for waste management evidencing that recycling should be a priority (Directive EC/2008). Although still not widely used, the vineyard produces a great quantity of vegetal byproducts and several studies have demonstrated that this biomass is also rich in polyphenolic compounds that could be exploited for different purposes and is affected by the extraction procedure [14,15,16,17]. Thus, *V. vinifera* cv. Aglianico leaves can be useful as a promising source of bioactive compounds giving a new perspective for the use of its by-product. The phenolic composition and biological activity of leaves was investigated in different cultivars of *V. vinifera* [18,19], but, to the best of our knowledge, this is the first study carried out on leaf extract from *V. vinifera* cv. Aglianico. Leaf secondary metabolites, mainly of phenolic origin, possess important beneficial properties for human health, including protective effect against the development and progression of pathological conditions such as cancer, aging, cardio-vascular problems and diabetes [11,12,20]. The oxidative stress is also associated with Alzheimer’s Disease (AD) and Parkinson’s Disease (PD), two neurodegenerative diseases characterized by cognitive disorders and memory loss. In AD, the “cholinergic hypothesis” is the most accepted theory, thus the inhibition of acetylcholinesterase (AchE) might alleviate the progressive deficiency of acetylcholine (ACh) levels that characterizes the pathology [21]. In PD, the dopamine tone is decreased and the disease is associated with tyrosinase-mediated neuronal damage. Although tyrosinase enzyme contributes to neuromelanin synthesis in the brain, with neuroprotective effects, its overexpression seems to play a pivotal role in generating oxidizing compounds that might exacerbate dopamine toxicity [22]. AD and PD progression can potentially slow down with the use of antioxidant compounds [23], as well as natural substances with anticholinesterase and antityrosinase activity. Borai and Rizk evaluated the neuroprotective and antioxidative potential of polyphenolic extract from *V. vinifera* leaves in alleviating aluminum chloride induced neurotoxicity in male rats. In fact, aluminum is a potent neurotoxic metal implicated in the neuropathogenesis of AD, because it induces a significant decrease of ACh content in the brain, along with a significant increment of AChE activity [24,25]. These promising *in vivo* results provide a concrete base in the field of natural products for discovery potential tyrosinase inhibitors.

Therefore, the objective of our research was to investigate the effect of different extraction processes on the polyphenolic profiles of *V. vinifera* L. cv. Aglianico leaf extracts and the antioxidant, anticholinesterase and antityrosinase activities.

## 2. Materials and Methods

### 2.1. Chemicals

Analytical grade methanol and acetonitrile were obtained from Merck (Darmstadt, Germany and Mollet del Vallés, Spain); 2,2-diphenyl-1-picryl hydrazyl (DPPH) in free radical form, 2,2′-azobis-(2-amidinopropane) dihydrochloride (AAPH), Trolox (6-hydroxy-2,5,7,8-tetramethylchroman-2-carboxylic), trizma hydrochloride (Tris-HCl), bovine serum albumin (BSA), Folin-Ciocalteu’s phenol reagent, *β*-carotene, linoleic acid, Tween 20, butylated hydroxytoluene (BHT, 2,6-bis (1,1-dimethylethyl)-4-methylphenol), 5,5′-dithio-bis (2-nitrobenzoic acid) (DTNB), acetylcholinesterase (AChE) from electric eel (type VI-s, lyophilized powder), acetylthiocholine iodide (ATCI), butyrylcholinesterase (BChE) from equine serum (lyophilized powder) and *S*-butyrylthiocholine chloride (BTCC), were purchased from Sigma (St. Louis, MO, USA and Steinheim, Germany). Fluorescein sodium was obtained via Fluka (Steinheim, Germany). Glacial acetic acid was obtained from Panreac (Barcelona, Spain). The standards of 38 phenolic compounds were purchased from Fluka, [5-methylfurfural, acetosiringone, benzoic acid, caffeic acid, caftaric acid, cinnamic acid, gallic acid, *p*-coumaric acid, (-)-epicatechin, quercetin glucoside, kaempferol-3-*O*-glucoside, homovanillic acid, ethyl gallate, *p*-hydroxybenzoic acid, quercetin and hydroxytyrosol], Sigma [(+)-taxifolin, (-)-epicatechin gallate, vanillic acid, 2-furfuraldehyde, ellagic acid, ferulic acid, gentisic acid, sinapic acid, syringic acid, (-)-catechin, (-)-epigallocatechin, ethyl vanillate, protocatechualdehyde, resveratrol and syringaldehyde], Merck, [vanillin, gentisin], Safc [ethyl vanillin and 5-hydroxymethyl-2-furaldeide] and Chromadex, [procyanidin-B1, procianydin-B2 and procyanidin-B3]. Double-distilled water (Millipore Co.) was used throughout.

### 2.2. Grape Leaves Collection

Grape leaves of *V. vinifera* L. (cv. Aglianico) were collected at the Martino wine company located in Venosa, Potenza (Basilicata Region). The plant material was kept at −20 °C. Leaves were milled manually to obtain a fine powder before the extraction.

### 2.3. Extraction of Antioxidant Compounds

Three different techniques were employed for the extraction of polyphenolic compounds from grape leaves—Soxhlet Extraction (SOX), Accelerated Solvent Extraction (ASE) and Ultrasound Assisted Extraction (UAE).

#### 2.3.1. Soxhlet Extraction (SOX)

SOX is a traditional method which has been used for many decades [26]. The finely ground plant material (25.40 g of dried leaves) was placed in a cellulose thimble with a porous bag made of filter paper, which is inserted on the top of the extraction chamber. When the solvent (ultrapure water:ethanol, 50:50) is heated, the vapors start to condense when they come into contact with the condenser and the solvent was collected in the collection flask. The condensed solvent will drip into the thimble containing the plant material. This process proceeded continuously and it was stopped when the solvent from the siphoned tube turned clear without any dissolved extraction material (about 6 h), meaning an exhausted extraction. The extract was then filtered and the solvent was removed by using a rotary evaporator.

#### 2.3.2. Accelerated Solvent Extraction (ASE)

Thermo Scientific Dionex ASE 150 Accelerated Solvent Extractor and 22 mL cells were used for the extraction of grape leaves of Aglianico. The extraction method was performed according to a previously described procedure [27,28,29], with slight modifications. The effect of two different temperatures of extraction, 40 °C (ASE 40) and 50 °C (ASE 50) was also tested. The assays were performed at 1600 psi for three cycles of 5 min each. For the extraction, the dried leaves of the Aglianico cultivar (30.29 g for ASE 40 and 26.17 g for ASE 50) were loaded and compacted in the cell and subjected to extraction with ethanol/water (50:50 *v/v*). The extracted solution was collected and filtered. The solvent was then removed via a rotary evaporator.

#### 2.3.3. Ultrasound Assisted Extraction (UAE)

The UAE method uses ultrasound waves for the extraction of natural compounds from grape leaves [30]. For the extraction by ultrasonic bath (Branson 1800 sonicator, frequency of 40 Hz and amplitude of 100%), 10.27 g of dried leaves of Aglianico were extracted using a mixture of 170 mL of ultrapure water and ethanol (50:50, *v/v*) as solvent. Extractions were performed in dark bottles at 30 °C for a period of 6 hours, the solvent has been changed every two hours with fresh one to facilitate the complete extraction. Finally, extracts were filtered and the solvent was removed with a rotary evaporator.

### 2.4. RP-HPLC-DAD Qualitative and Quantitative Analysis of Phenolic Compounds

The LC analysis of phenols was performed using an Agilent Series 1100 system equipped with a quaternary pump (Series 1100 G1311A), automatic injector (Series 122 1100 G1313A) and degasser on line (Series 1100 G1379A). A UV/Vis diode detector (Series 1100 G1315B) coupled to a Chemstation HP A.10.02 (HP/Agilent) was used for detection. The column was a Merck LiChroCART RP-18 250-4 Superspher 100 RP-18, pore size 5 µm (250 mm × 4 mm), protected by precolomn Merk RP-18 4.6 × 12.5 mm. Samples were filtered before injection through a Whatman filters cellulose acetate membrane, pore size 0.45 µm and the diameter size 25 mm. The chromatographic conditions have been previously described [31]. Two different solvents were used as a mobile phase—A (glacial acetic acid/water, pH 2.65), B (20% A + 80% acetonitrile) programmed in a gradient as follows—0 min (100% A); 5 min (98% A + 2% B); 10 min (96% A + 4% B); 15 min (90% A + 10% B); 30 min (80% A + 20% B); 35 min (70% A + 30% B); 40 min (100% B); 45 min (100% A); 60 min (100% A). The injection volume was 50 µL. The flow rate was 1.5 mL·min^−1^ and the temperature was set at 40 ºC. The identification of each compound was obtained according the retention time and the UV-Visible spectra of the corresponding standard compound. Standard compounds were solubilized in methanol at a concentration of 1.5 mg/mL, used as stock solution and subsequently diluted for the calibration curve. Quantification analysis was performed by external calibration with respective standards at 280 nm for hydroxybenzoic acids, 320 nm for hydroxycinnamic acids and 365 nm for flavonoids, in accordance with the maximum absorbance of each compound. Results of quantitative analysis were expressed as mg of compound/kg of dried extract [32].

### 2.5. Total Phenolic Content (TPC)

All extracts were used to test the total phenolic content (TPC) by using the Folin-Ciocalteu assay [33] and 75 μL of the diluted extract and 425 μL of distilled water was added to 500 μL F-C reagent and 500 μL of Na_2_CO_3_ (10% *w/v*). Sodium carbonate is added to alkalise the system obtaining a pH value about 10. The solution was mixed and incubated for 1 h in the dark at room temperature. After incubation, the absorbance was measured at 723 nm using a UV-Vis spectrophotometer SPECTROstar^Nano^ (BMG Labtech). A standard calibration curve was prepared using different concentrations of gallic acid and results were expressed as µg of Gallic Acid Equivalents (GAE)/g of extract, using the regression equation between gallic acid standards and absorbance (y =0.0871x −0.0282; R² = 0.9997). For each sample, three replicate assays were performed.

### 2.6. Antioxidant Activity

The antioxidant activity was determined by different spectrophotometric techniques which are described below.

#### 2.6.1. DPPH Method

Free radical scavenging activity of the extracts was evaluated using the DPPH method [34] with some modifications. This method evaluates the quenching ability of our extract toward the DPPH radical, by spectrophotometric monitoring of the following reaction:

DPPH·+R:H →DPPH−H+R,

where R:H represents an antioxidant. For each extract, a dilution series was prepared (final concentration ranging from 20-50 µg/mL of leaves extracts). In each well, 50 µL of the sample or solvent for the blank were added to 150 µL of the DPPH solution (120 µM). DPPH scavenging activity was monitored at 515 nm using a UV-Vis spectrophotometer (Synergy HT, Biotek®) at 0 min and after 20 min, when the reaction reached the equilibrium. The antioxidant activity was expressed as IC_50_ (µg/mL), the concentration of substrate that is required to scavenge 50% of DPPH free radicals [35]. To calculate the percentage of inhibition of the radical, the absorbance values (A) at equilibrium (T = 20 min) of both the control (CT) and the sample (S) were measured:

% inhibition = [(A_CT_ − A_S_)/A_CT_) *100]. (1)

#### 2.6.2. Oxygen Radical Antioxidant Capacity (ORAC) assay

The ORAC assay was based on a previously reported method with slight modifications [36]. It is based on in situ generation of peroxyl free radicals by 2,2-azobis(2-methylpropionamidine) dihydrochloride (AAPH). They interact with oxidable fluorescent probe (F·), changing the fluorescence intensity. In the presence of antioxidants (ArOH), the fluorescence decay is inhibited, as is illustrated in the following chemical equation:

F+ArOH →FH+ArO

The analytical procedure was as follows—50 µL of sample or Trolox was added to 100 µL of Fluorescein (1.5 µM) and 50 µL of 2,2′-azobis (2-amidinopropane) dihydrochloride (AAPH, 15 mM). For the blank, 50 µL of phosphate buffer (75 mM, pH 7.4) was added to 100 µL of Fluorescein and 50 µL of AAPH, whereas for the control, 50 µL of phosphate buffer was added to 100 µL of Fluorescein. The plate was incubated for 15 min at room temperature after addition of AAPH. Fluorescence (the excitation wavelength was set at 490 nm; the emission wavelength was 515 nm) was calculated every 5 min for 90 min at 37 °C, until it approximately decreases to 0 or to a value less than 5% of the initial value. Measurements were taken in duplicate in a multi-220 detector microplate reader (Synergy HT, Biotek®, Winooski, VT, USA). Trolox was used as a calibration standard (0.5–9.5 µM). The results were calculated as ORAC values indicating the differences between the blank and the sample areas under the fluorescein decay curve [37].

The equation used is the following:

ORAC value = 20 × K × [(S_sample_ − S_blank_) / (S_trolox_ − S_blank_)] (2)

where 20 is the concentration of Trolox; K is the dilution factor of sample; S is the area under the curve of the decrease of the fluorescein, the sample, the Trolox or the blank.

The results are expressed as μmol Trolox equivalents (TE)/mg of extract.

#### 2.6.3. *β*-Carotene Bleaching Assay (BCB)

The antioxidant activity was also evaluated by *β*-carotene bleaching assay (BCB) [38]. *β*-Carotene (0.2 mg) was dissolved in 0.2 mL of chloroform and then the chloroform was removed by rotary evaporator at room temperature. The *β*-carotene solution was added to a flask together with linoleic acid (20 mg) and Tween 20 (200 mg) and finally oxygenated distilled water (50 mL) was added and mixed well. Aliquots of the emulsion (950 µL) were mixed into different test tubes with 50 µL of sample (the final concentration for all tested samples was 200 µg/mL) or solvent as a blank. Butylhydroxytoluene (BHT) was used as positive control. This emulsion solution (250 μL) was transferred to a 96-well microplate. Then, the microplate was immediately placed at 50 °C for 3 h and the absorbance was measured at 470 nm, using a spectrophotometer, every 30 min—at 0′, 30′, 60′, 90′, 120′, 150′ and 180′ until the color of control sample has changed. Results were expressed as percentage of Antioxidant Activity (AA%) and it was calculated using the following equation:

(3) $$\left(AA\%\right)=\left(1-\frac{Abs\;sample_{T_{0^{\prime }}}-Abs\;sample_{T_{180^{'}}}}{Abs\;blank_{T_{0^{\prime }}}-Abs\;blank_{T_{180^{'}}}}\right)\times 100$$

where *Abs sample T_0′_* and *Abs blank T_0′_* are the absorbance before the incubation of the extract and the blank (without extract), respectively and *Abs sample T_180_*_′_ and *Abs blank T_180_*_′_ are the absorbance at 180 min of incubation of extract and the blank, respectively.

### 2.7. Acetylcholinesterase (AChE) and Butyrylcholinesterase (BChE) Inhibitory Activity

The inhibition of AChE activity was determined based on Ellman’s method, as previously reported [38]. The enzyme activity is measured by spectrophotometric detection (405 nm) of the increase of yellow color produced from thiocholine when it reacts with 5,5′-dithio bis-2 nitrobenzoate ions (DTNB). For the analysis, 25 µL of acetylthiocholine iodide (15 mM), 125 µL of DTNB (3 mM), 50 µL of buffer B (50 mM Tris-HCl, pH 8 containing 0.1% bovine serum albumin) and 25 µL of each test sample solution at the different concentrations were mixed. The reaction was started by adding 25 µL of 0.18 U/mL AChE. The absorbance was measured at 405 nm kinetically during 2 min. Negative control absorbance (Buffer A, 50 mM Tris-HCl, pH 8) was also measured. The BChE inhibition assay was performed in a similar way [38] using 25 µL of 15 mM *S*-butyrylthiocholine chloride as substrate and 0.10 U/mL of BChE as enzyme. Samples were evaluated at different concentrations. In the same way, negative control absorbance (Buffer A) was also recorded. The results were expressed as percentage of inhibition and calculated as follows [39]:

% inhibition = ((Abs_sample_ − Abs_negative control_) × 100 − 100)^−1^ (4)

Galantamine, dissolved in Buffer A, was used as positive control for both assays.

### 2.8. Tyrosinase Inhibitory Activity

The inhibition of tyrosinase was performed by L-DOPA in vitro assay [40]. A reaction mixture, containing 125 mL of phosphate buffer (50 mM, pH 6.8), 25 μL of standard or extracts at different concentrations and 50 mL of tyrosinase (50U/mL) was incubated at 37 °C for 15 min. Then, the L-DOPA substrate (50 μL) was added to the mixture and the reaction was monitored for 10 min at 475 nm. The experiment was made in triplicate. The results were expressed as IC_50_ value, a concentration giving 50% inhibition of tyrosinase activity, determined by interpolation of concentration-response curves. Kojic acid was used as a positive control.

### 2.9. Statistical Analysis

Analysis of variance was performed to assess the statistically significant differences among samples, for the polyphenolic content of grape leaves of Aglianico cultivar, at a confidence level of 95% [41]. Difference on the mean values was assessed by the Tukey test at a significance level of *p* < 0.05. To compare the results between the different methods, the Relative Antioxidant Capacity Index (RACI) was calculated [42].

## 3. Results and Discussion

### 3.1. Vitis vinifera cv. Aglianico Leaf Extraction

Extraction yield is a quantitative representation of the efficiency of the extraction process to recover natural compounds from the plant tissues [43]. Parameters, such as extraction technique, solvent, temperature and time, as well as the chemical nature of the sample [44], affect the extractive yield. In this work, leaves from *Vitis vinifera* L. cv. Aglianico were extracted with the same solvent (water:ethanol 50:50) but by using different solid–liquid extraction techniques. All extracts were dried by rotary evaporator and yield extraction was calculated. The results of extractive yields were expressed as the percentage of the weight of the crude extract *vs.* raw material. Table 1 shows the different extractive capacities of each technique. Extractive yields ranged from 6.41 ± 0.52% to 30.45 ± 2.32%. 

The choice of this mix of solvents allows us to perform an eco-sustainable extraction, obtaining good extractive yields. Our results, indeed, are comparable with these of Matloub [45] who achieved yields ranging from 12.10 ± 1.81% to 44.99 ± 1.19%, by using different mix of acetone and methanol. These toxic solvents are more expensive and require higher disposal costs, at the expense of the environment. ASE conditions (temperature, cycles and solvent) have been selected basing on the results obtained by previous studies [20] and optimized in order to obtain the complete extraction of metabolites. However, ASE system reported the lowest extractive yield at both used temperature (40 and 50°C), whereas the highest extraction yield was observed in SOX extract. The fact that the sample is repeatedly in contact with fresh solvent and the high temperature reached by SOX method, could be the reason of the higher content of secondary metabolites extracted from leaf material. In fact, temperature is an important parameter contributing to the extraction yield. Usually, elevated temperatures lead to the improved extraction efficiencies [46] but they can present the inconvenience of the degradation of thermo labile compounds.

### 3.2. Identification and Quantification of Phenolic Compounds

**Figure 1:**
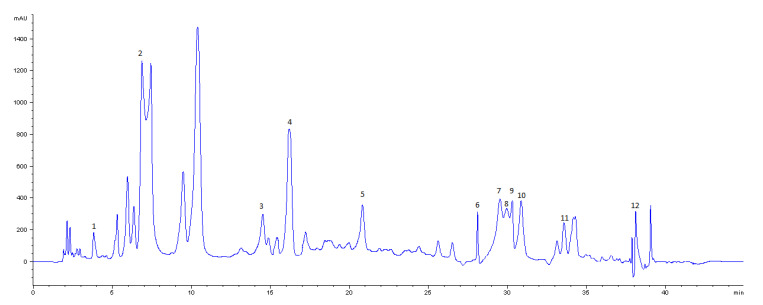
HPLC chromatogram of Soxhlet (SOX) leaf extract of Vitis vinifera cv. Aglianico (320 nm). Peaks: 1, gallic acid; 2, caftaric acid; 3, (+)-catechin; 4, caffeic acid; 5, benzoic acid; 6, rutin; 7, quercetin-3-O-galactoside; 8, quercetin-3-O-glucoside; 9, quercetin -3-O-glucuronide; 10, quercetin-3-O-glycoside; 11, kaempferol-3-O-glucoside; 12, quercetin.

The identification and quantification of phenolic compounds in the leaves of Aglianico was carried out using the HPLC-DAD method. As reported in Figure 1, a total of 9 phenolic compounds [gallic acid, (+)-catechin, benzoic, caftaric and caffeic acids, rutin, quercetin, quercetin-3-*O*-glucoside and kaempferol-3-*O*-glucoside] were identified and quantified in our samples (Table 2). 

Moreover, other 3 flavonols could be presumably identified on the basis of the literature [quercetin-3-*O*-galactoside, quercetin-3-*O*-glucuronide and quercetin-3-*O*-glycoside]. SOX extract of leaf tissue allowed the identification of these phenolic compounds. However, gallic and caftaric acid, quercetin-3-*O*-glucoside and quercetin were not present in ASE 40, ASE 50 and UAE.

For quantification analysis, pure compounds were used as standards. All the analyzed compounds, detection wavelengths, maximum absorptions, retention times and concentrations are listed in Table 2. Their total amount (expressed as mean ± standard deviation /Kg of extract) ranged from 20,640.62 ± 200.36 mg /Kg in SOX extract to 11383.15 ± 116.06 mg /Kg in ASE 40 extract.The identified compounds were in accordance with other works already published on different leaf extracts of *V. vinifera* L. [18,47]. The hydroxycinnamic and hydroxybenzoic acids are normally found in grape peels, however leaves can be also considered a new source of phenolic acid [48,49]. Among hydroxybenzoic acids, gallic acid was found in SOX extract (159.91 ± 1.54 mg/Kg). Moreover, benzoic acid is present in all samples with a mean value of 327.03 mg/Kg; ASE 40 was the extract with the highest content of benzoic acid (452.83 ± 2.76 mg/Kg). In comparison, the hydroxycinnamic acid content was higher than that of hydroxybenzoic acids. The most abundant hydroxycinnamic acid was caftaric acid; as shown in Table 2, the highest content was shown in UAE extract (6047.84 ± 41.30 mg/Kg). Guidoni et al. [50] identified tartaroyl esters of *trans*-caffeic acid and *trans*-coumaric acid as the principal hydroxycinnamic acid derivatives in grapevine leaves. The content of flavan-3-ols in the leaf extracts of Aglianico grapevine was higher than other studies [51]. In particular, the content of (+)-catechin in SOX was 1176.00 ± 17.32 mg/Kg. Concerning the flavonols and in accordance with literature [51], the amount of quercetin derivatives in leaves was higher than in the kaempferol derivatives. Weber (1993) detected different *O*-glycosides of quercetin and kaempferol in grapevine leaves [52]. Consequently, we could suppose, with good approximation, that the peaks 7, 9 and 10 corresponded to some O-glycoside derivatives, probably quercetine-3-O-galactoside, -3-*O*-glucuronide and -3-*O-*glycoside, respectively, basing on literature evidence [52]. In the case of kaempferol glycosides, it corresponded to the -3-*O*-glucoside. Quercetin-3-*O*-glucoside is present only in SOX sample in huge quantity (2352.24 ± 41.34 mg/Kg). In relation to the aglycones, quercetin was found at low levels and it was detected only in SOX sample with 490.71 ± 0.63 mg/Kg.

### 3.3. Total Polyphenol Content and Antioxidant Activity

**Figure 2:**
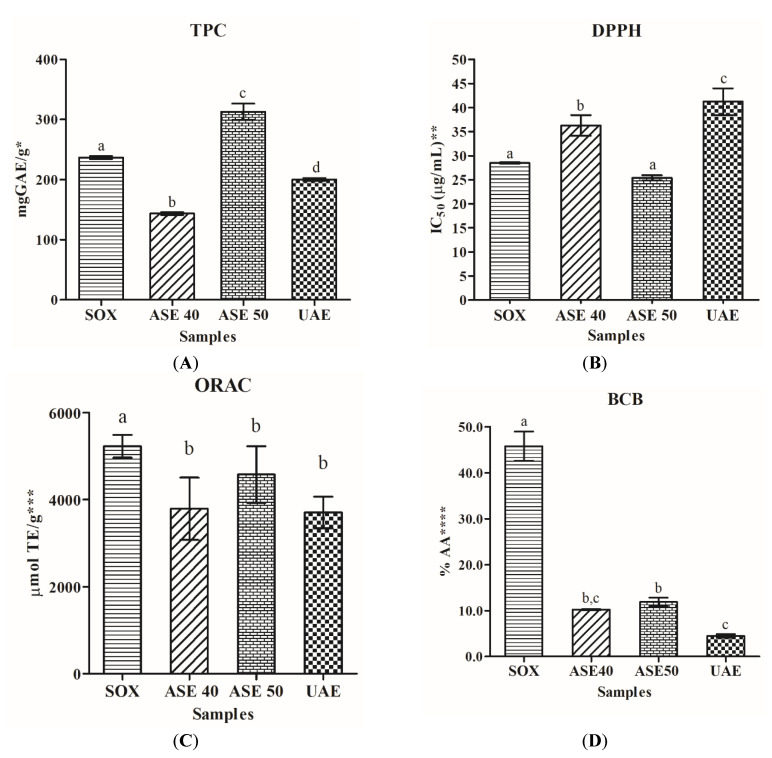
Samples are extracts obtained by Soxhlet extractive technique (SOX), Accelerated Solvent Extraction at 40 °C (ASE 40), Accelerated Solvent Extraction at 50 °C (ASE 50) and Ultrasound-assisted extraction (UAE). Histograms of (**A**) Total Phenolic Content (TPC); (**B**) DPPH method; (**C**) Oxygen Radical Antioxidant Capacity (ORAC) assay; (**D**) *β*-Carotene Bleaching assay (BCB); data are expressed as means ± standard deviation from three experiments; * mg GAE/g = mg of Gallic Acid Equivalents per gram of dried sample; ** IC_50_ (μg/mL) = concentration of the sample able to scavenge 50% DPPH radical; *** µmol TE/g = µmol of Trolox Equivalents per gram of dried sample; **** % AA = percentage of Antioxidant Activity at final sample concentration of 0.2 mg/mL; different superscripts in the same row indicate significant difference (*p* < 0.05).

The evaluation of Total Polyphenol Content (TPC) was carried out using Folin-Ciocalteu method and the results were expressed as mg Gallic Acid Equivalents (GAE)/g of dried extract [33]. The total phenolic content of extracts ranged from 143.37 ± 2.33 to 312.78 ± 13.56 mg GAE/g in ASE 40 and ASE 50 extracts, respectively. These results are higher than these reported by Ferhi, Santaniello, Zerizer, Cruciani, Fadda, Sanna, Dore, Maioli and D’hallewin [20], confirming that our attempt to optimize extraction condition has been successful. High TPC value was also observed in SOX extract (236.43 ± 2.62 mg GAE/g), as shown Figure 2A. Different phenolic contents of grape leaves are reported in the literature. Uysal et al. [53] showed that the total phenolic content of leaf extract ranged from 60.14 ± 4.33 and 64.66 ± 0.35 mg GAE/g by a Soxhlet apparatus using methanol and water respectively. Once again, our choice of solvents is not only cheaper and more eco-friendly but also provides better results for the recovery of polyphenols.

The free radical-scavenging activity of extracts is consistent with TPC, the highest radical scavenging activity was indeed observed for ASE 50 extract with IC_50_ value of 25.40 ± 0.54 µg/mL (Figure 2B), followed by SOX extract. Both extracts reported high content of polyphenols that can justify the good radical scavenging activity. However, our results are better than those previously reported for grape leaf ASE hydroethanolic extract (0.09 ± 0.32 mg/mL) [20]. Concerning the ORAC (expressed as µmol of Trolox equivalent (TE)/g of extract), the values ranged from 3702.71 ± 366.90 to 5227.09 ± 261.32 µmol TE/g (Figure 2C) and SOX extract reported the highest ORAC value. It is important to highlight that the leaves of Aglianico presented higher ORAC values in comparison with grape leaf extracts reported in other studies (ranging from 1.52 to 2.55 µmol Trolox equivalents/mg of extract) [51].

Lipid peroxidation inhibition, carried out by *β*-carotene bleaching (BCB) assay, showed that all extracts exhibited moderate *β*-carotene bleaching inhibition activity, lower than 50% at a final sample concentration of 0.2 mg/mL. In fact, results ranged from 4.50 ± 0.40 to 45.80 ± 3.20%AA in the UAE and SOX extracts, respectively (Figure 2D). Several studies showed no correlation between TPC and BCB [54]. In this context, if TPC gives an indication of the levels of both lipophilic and hydrophilic compounds, BCB, in contrast, only gives an indication of the levels of lipophilic compounds [55]. ASE 40 and ASE 50 extracts have similar value of lipid peroxidation inhibition in spite of very large differences in TPC. This implies the presence of approximately similar amounts of lipophilic antioxidants in both samples.

**Figure 3:**
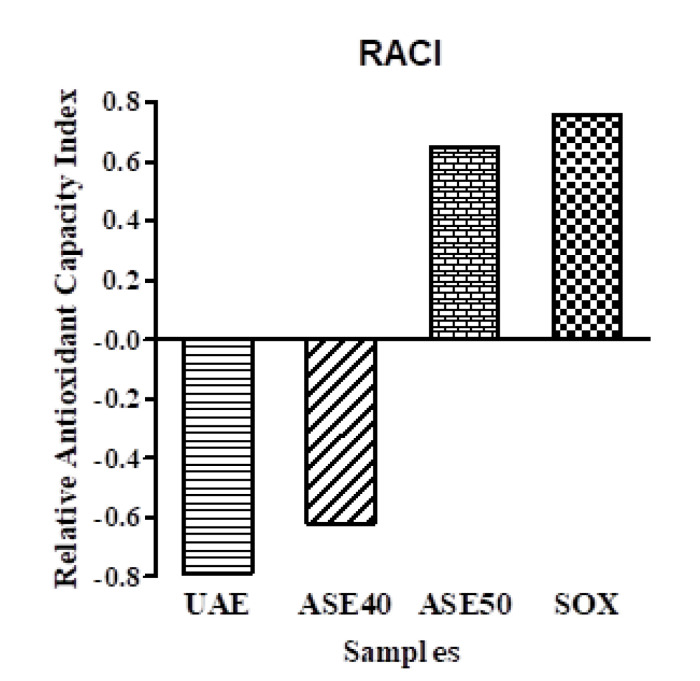
Relative Antioxidant Capacity Index (RACI) values obtained comparing TPC, DPPH, ORAC and BCB results of Aglianico leaf extracts. Samples are extracts obtained by Soxhlet extractive technique (SOX), Accelerated Solvent Extraction at 50 °C (ASE 50) and Ultrasound Assisted Extraction (UAE).

A new concept, Relative Antioxidant Capacity Index (RACI), was applied, integrating antioxidant capacity data determined by several methods [42]. To compare the antioxidant capacity of extracts derived from different chemical methods, results of TPC, DPPH, ORAC and BCB were used to calculate the RACI. Data of relative antioxidant activity were represented in Figure 3. According to obtained results, the leaf extract obtained by Soxhlet technique showed the highest RACI value of 0.76, followed by ASE 50 (0.65).

### 3.4. Inhibitory Activity Against Acetylcholinesterase (AChE) and Butyrylcholinesterase (BChE)

AChE and BChE are two enzymes that differ genetically, structurally and for their typical kinetics. AChE is a hydrolase that plays a key role in cholinergic transmission by catalyzing the rapid hydrolysis of the neurotransmitter acetylcholine (Ach) [56]. The BChE is plentiful in plasma and in different parts of human body; it has a similar protein structure to that of AChE and for this reason it is called the sister enzyme. When the AChE is inhibited, BChE can substitute the absent activity of AChE; then the inhibition of BChE is an important strategy for AD research [57]. Several studies recently supported that different plant extracts and active compounds have anticholinesterase activity [23,38,57]. The enzymatic inhibition activity was determined in leaf extracts of Aglianico at different concentrations. Not all samples reached the IC_50_ value at tested concentration; for this reason, AChE inhibitory activity was represented as the percentage of inhibition at the common concentration of 125 µg/mL of leaf extract. ASE 50 shown a great good AChE inhibitory activity (Table 3) with a 50.65 ± 3.12% of inhibition (IC_50_ = 107.16 ± 8.12 µg/mL). In contrast to our results, other paper using methanolic extract of leaves did not report AChE activity [53]. This difference can be ascribed to the different extraction procedure that allowed us to extract different compounds, increasing the activity of the phytocomplex. In the BChE assay, the leaf extracts of Aglianico displayed a less activity than the AChE (Table 3) and SOX showed 43.85 ± 2.17% of BChE inhibition at 125 µg/mL (IC_50_ = 171.24 ± 12.12 µg/mL). Although BChE inhibition is minor, it is very important due to the possibility of BChE to replace AChE in hydrolyzing brain acetylcholine, mainly in advanced stages of AD. Thus, these natural double inhibitors should provide an efficient treatment in AD patients [56]. 

### 3.5. Inhibitory Activity Against Tyrosinase

Tyrosinase is a copper enzyme that catalyzes the oxidation of L-tyrosine to 3,4 dihydroxyphenylalanine (DOPA). This is the rate-limiting step in the melanogenesis, a process that takes place in melanocytes and results in the synthesis of melanin pigments [30]. Moreover, the tyrosinase enzyme plays a pivotal role in neuromelanin production. This pigment, in human brain demonstrated neuroprotective properties but its overexpression, mainly in PD patients, is associated with neuronal damage, as extensively reported in the literature [22]. Thus, given the importance of tyrosinase inhibitor’s discovery and development, our research fits well with this hot topic. Between the various types of tyrosinase inhibitors (competitive, uncompetitive, mixed type and noncompetitive), kojic acid shows a typical competitive inhibitory effect on tyrosinase [30], thus it has been selected as positive control (IC_50_ = 3.9 ± 0.49 μg/mL). The highest inhibition values were obtained by UAE (IC_50_ = 293.2 ± 25.6 μg/mL) and SOX (IC_50_ = 302.5 ± 38.3 μg/mL) (Table 4). To the best of our knowledge, this is one of the first reports of antityrosinase activity of grape leaf extracts. The efficacy of our extracts were revealed to be considerably higher with respect to the previously reported activity of the aqueous extract of Vitis vinifera (IC_50_ = 3.84 mg/mL) [30]. The better inhibitory activity showed by our extract should be due to the optimization of extraction conditions. Most of all, ethanol addition may have allowed the recovery of a high amount of secondary metabolites with antityrosinase activity. Among them, gallic acid, caffeic acid and quercetin are detectable only in SOX extract. The first inhibits the oxidation of L-DOPA catalyzed by tyrosinase [58]; the second can act as a suicide substrate of the enzyme [59]. Quercetin and kaempferol, thanks to the 3-hydroxy-4-keto moiety, can chelate copper in the active site of the enzyme, leading to a competitive and irreversible inhibition of enzyme [58]. However, the *O*-glycoside derivatives seem to be not active [58]. In accordance with the higher antityrosinase activity showed by UAE and SOX extracts, caftaric acid, which is a proven competitive inhibitor of tyrosinase [60], can be found mostly in these two extracts.

## 4. Conclusions

Grape leaves possess great health-promoting properties that have allowed their use in traditional medicine. The interest in food by-products is currently rising, due to the cost-effectiveness of these matrices as sources of natural bioactive compounds. Grape leaves, indeed, were revealed to be particularly rich in polyphenolic compounds, with great antioxidant activity. Moreover, in this study, *Vitis vinifera* L. cv. Aglianico, a variety of red grape cultivated in Southern Italy, was investigated for the first time for its phytochemical profile and antioxidant, anticholinesterase and antityrosinase activity. The application of eco-sustainable techniques showed good results in terms of the extraction of bioactive compounds, mainly for SOX extract. This method, thanks to the high temperature reached and the prolonged contact with fresh solvent, allowed the recovery of a great amount of phenolic compounds, as demonstrated by results of Folin-Ciocalteu assay. The HPLC-DAD analysis, indeed, led to the identification and quantification of 9 phenolic compounds with inhibitory properties against cholinesterase and tyrosinase enzymes. This promising evidence promotes the investigation of the application of leaf extracts in the treatment of neurodegenerative diseases, giving by-products a boost toward a definitive seal of approval.

## Figures and Tables

**Table 1:** Extraction parameters to recovery the bioactive compounds from grape leaves of *Vitis vinifera* L. (cv. Aglianico).

Method of Extraction	Solvent of Extraction	G	Temperature	Time of Extraction	Extraction Yield (%)
Soxhlet extraction (SOX)	Ultrapure water and ethanol, 50:50	25.40 g	100 °C	6 h	30.45 ± 2.32 ^b^
Ultrasound Assisted Extraction (UAE)	Ultrapure water and ethanol, 50:50	10.27 g	30 °C	6 h	13.81 ± 1.13 ^c^
Accelerated Solvent Extraction (ASE 40)	Ultrapure water and ethanol, 50:50	30.29 g	40 °C	static time 5 min × 3 cycles	6.44 ± 0.48 ^a^
Accelerated Solvent Extraction (ASE 50)	Ultrapure water and ethanol, 50:50	26.17 g	50 °C	static time 5 min × 3 cycles	6.41 ± 0.52 ^a^

Extractions were repeated in triplicate and expressed as mean ± standard deviation. Significant differences (*p* < 0.05) are highlighted with different superscript letters (a, b, and c).

**Table 2:** Quantification of compounds in *Vitis vinifera* (cv. Aglianico) leaf extracts.

Peak	Analyte	RT (min)	λmax	SOX	ASE 40	ASE 50	UAE
1	Gallic acid	3.9	280	159.91 ± 1.54 ^a^	Nd	Nd	Nd
2	Caftaric acid	6.7	320	5706.97 ± 77.20 ^a^	3651.71 ± 52.91 ^b^	4075.15 ± 23.40 ^c^	6047.84 ± 41.30 ^d^
3	(+)-Catechin	14.7	280	1176.00 ± 17.32 ^a^	300.52 ± 0.41 ^b^	331.72 ± 0.52 ^c^	682.81 ± 2.72 ^d^
4	Caffeic acid	16.3	320	557.31 ± 2.68 ^a^	Nd	Nd	Nd
5	Benzoic acid	21.8	280	131.23 ± 4.89 ^a^	452.83 ± 2.76 ^b^	315.78 ± 1.85 ^c^	408.27 ± 0.00 ^d^
6	Rutin	28.1	365	205.69 ± 15.34 ^a^	210.64 ± 7.20 ^a^	192.23 ± 18.89 ^a^	319.75 ± 1.49 ^b^
7	Quercetin-3-*O*-galactoside	29.5	365	2938.56 ± 6.79 ^a^	1976.24 ± 20.78 ^b^	2003.21 ± 16.80 ^b^	2449.91 ± 3.43 ^c^
8	Quercetin-3-*O*-glucoside	29.9	365	2352.24 ± 41.34 ^a^	Nd	Nd	Nd
9	Quercetin -3-*O*-glucuronide	30.3	365	2893.19 ± 21.59 ^a^	2009.87 ± 5.99 ^b^	2023.01 ± 3.45 ^b^	2465.27 ± 3.43 ^c^
10	Quercetin-3-*O-*glycoside	30.8	365	3362.17 ± 4.94 ^a^	2219.28 ± 1.60 ^b^	2450.58 ± 23.26 ^c^	3150.82 ± 10.29 ^d^
11	Kaempferol-3-*O*-glucoside	33.6	365	666.64 ± 6.10 ^a^	562.06 ± 24.41 ^b^	535.27 ± 24.99 ^b^	572.65 ± 10.39 ^b^
12	Quercetin	38.1	365	490.71 ± 0.63 ^a^	Nd	Nd	Nd
	TOTAL			20640.62 ± 200.36	11383.15 ± 116.06	11926.95 ± 96.36	16097.32 ± 73.05

Results are expressed as mean ± standard deviation as mg of single standard/Kg of extract; RT = retention time; λmax = wavelength of maximum absorbance; samples are extracts obtained by Soxhlet extractive technique (SOX), Accelerated Solvent Extraction at 40 °C (ASE 40), Accelerated Solvent Extraction at 50 °C (ASE 50) and Ultrasound Assisted Extraction (UAE); nd = not detected; different superscript letters (a, b, c and d) denote statistically significant differences in the same column (*p* < 0.05).

**Table 3:** Results of inhibitory activity against acetylcholinesterase (AChE) and butyrylcholinesterase (BChE) enzymes.

Samples	% Inhibition (at 125 μg/mL)		IC_50_ (µg/mL) *	
	AChE	BChE	AChE	BChE
**Galantamine**	95.82 ± 1.60	97.55 ± 1.60	1.53 ± 0.11	1.85 ± 0.08
**SOX**	31.29 ± 0.09 ^a^	43.85 ± 2.17 ^a^	Nd	171.34 ± 12.12
**ASE 40**	34.60 ± 2.05 ^a^	4.19 ± 0.35 ^b,c^	Nd	Nd
**ASE 50**	50.65 ± 3.12 ^b^	15.65 ±0.21 ^b,d^	107.16 ± 8.12	Nd
**UAE**	25.52 ± 0.61 ^a^	11.8 ± 1.6 ^c,d^	Nd	Nd

Samples are Galantamine (positive control), extracts obtained by Soxhlet extractive technique (SOX), Accelerated Solvent Extraction at 50 °C (ASE 50) and Ultrasound Assisted Extraction (UAE); data are expressed as means ± standard deviation from three experiments; *: IC_50_ (μg/mL) is the concentration of the sample able to inhibit 50% enzymatic activity; different superscript letters (a, b, c, and d) denote statistically significant differences in the same column (*p* < 0.05).

**Table 4:** Results of inhibitory activity against tyrosinase enzyme.

Samples	IC_50_ (µg/mL) *
	Tyrosinase
**Kojic acid**	3.9 ± 0.49 ^a^
**SOX**	302.5 ± 38.30 ^b^
**ASE 40**	568.7 ± 1.801 ^c^
**ASE 50**	727.1 ± 48.23 ^d^
**UAE**	293.2 ± 25.60 ^b^

Samples are kojic acid (positive control), extracts obtained by Soxhlet extractive technique (SOX), Accelerated Solvent Extraction at 50 °C (ASE 50) and Ultrasound Assisted Extraction (UAE); data are expressed as means ± standard deviation from three experiments; *: IC_50_ (μg/mL) is the concentration of the sample able to inhibit 50% enzymatic activity; different superscript letters (a, b, c and d) denote statistically significant differences in the same column (*p* < 0.05).

## References

[1] FAOSTAT. http://www.fao.org/faostat/en/#data/QC.

[2] Gabaston J., El Khawand T., Waffo-Teguo P., Decendit A., Richard T., Mérillon J.-M., Pavela R. (2018). Stilbenes from grapevine root: A promising natural insecticide against *Leptinotarsa decemlineata*. J. Pest. Sci..

[3] Dukić D., Mašković P., Moračanin S.V., Kurćubić V., Milijašević M., Babić J. Conventional and unconventional extraction methods applied to the plant, *Thymus serpyllum* L.. Proceedings of the IOP Conference Series: Earth and Environmental Science.

[4] Russo D., Faraone I., Labanca F., Sinisgalli C., Bartolo M., Andrade P.B., Valentao P., Milella L. (2019). Comparison of different green-extraction techniques and determination of the phytochemical profile and antioxidant activity of Echinacea angustifolia L. extracts. Phytochem. Anal..

[5] De la Cerda-Carrasco A., López-Solís R., Nuñez-Kalasic H., Peña-Neira Á., Obreque-Slier E. (2015). Phenolic composition and antioxidant capacity of pomaces from four grape varieties (*Vitis vinifera* L.). J. Sci. Food Agric..

[6] Cafaro C., Bonomo M.G., Guerrieri A., Crispo F., Ciriello R., Salzano G. (2016). Assessment of the genetic polymorphism and physiological characterization of indigenous *Oenococcus oeni* strains isolated from Aglianico del Vulture red wine. Folia Microbiol..

[7] De Nisco M., Manfra M., Bolognese A., Sofo A., Scopa A., Tenore G.C., Pagano F., Milite C., Russo M.T. (2013). Nutraceutical properties and polyphenolic profile of berry skin and wine of *Vitis vinifera* L. (cv. Aglianico). Food Chem..

[8] Rinaldi A., Jourdes M., Teissedre P., Moio L. (2014). A preliminary characterization of Aglianico (*Vitis vinifera* L. cv.) grape proanthocyanidins and evaluation of their reactivity towards salivary proteins. Food Chem..

[9] Rinaldi A., Villano C., Lanzillo C., Tamburrino A., Jourdes M., Teissedre P.-L., Moio L., Frusciante L., Carputo D., Aversano R. (2017). Metabolic and RNA profiling elucidates proanthocyanidins accumulation in Aglianico grape. Food Chem..

[10] Xia E.-Q., Deng G.-F., Guo Y.-J., Li H.-B. (2010). Biological activities of polyphenols from grapes. Int. J. Mol. Sci..

[11] Quideau S., Deffieux D., Douat-Casassus C., Pouysegu L. (2011). Plant polyphenols: Chemical properties, biological activities, and synthesis. Angew. Chem. Int. Ed..

[12] Pandey K.B., Rizvi S.I. (2009). Plant polyphenols as dietary antioxidants in human health and disease. Oxid. Med. Cell. Longev..

[13] Gabriele M., Gerardi C., Lucejko J.J., Longo V., Pucci L., Domenici V. (2018). Effects of low sulfur dioxide concentrations on bioactive compounds and antioxidant properties of Aglianico red wine. Food Chem..

[14] Alexandru L., Binello A., Mantegna S., Boffa L., Chemat F., Cravotto G. (2014). Efficient green extraction of polyphenols from post-harvested agro-industry vegetal sources in Piedmont. C. R. Chim..

[15] Dani C., Oliboni L., Agostini F., Funchal C., Serafini L., Henriques J., Salvador M. (2010). Phenolic content of grapevine leaves (*Vitis labrusca* var. Bordo) and its neuroprotective effect against peroxide damage. Toxicol. In Vitro.

[16] Lima M.R., Felgueiras M.L., Cunha A., Chicau G., Ferreres F., Dias A.C. (2017). Differential phenolic production in leaves of *Vitis vinifera* cv. Alvarinho affected with esca disease. Plant Physiol. Biochem..

[17] Crupi P., Dipalmo T., Clodoveo M.L., Toci A.T., Coletta A. (2018). Seedless table grape residues as a source of polyphenols: Comparison and optimization of non-conventional extraction techniques. Eur. Food Res. Technol..

[18] Fernandes F., Ramalhosa E., Pires P., Verdial J., Valentão P., Andrade P., Bento A., Pereira J.A. (2013). *Vitis vinifera* leaves towards bioactivity. Ind. Crops Prod..

[19] Katalinić V., Generalić I., Skroza D., Ljubenkov I., Teskera A., Konta I., Boban M. (2009). Insight in the phenolic composition and antioxidative properties of *Vitis vinifera* leaves extracts. Croat. J. Food Sci. Technol..

[20] Ferhi S., Santaniello S., Zerizer S., Cruciani S., Fadda A., Sanna D., Dore A., Maioli M., D’hallewin G. (2019). Total phenols from grape leaves counteract cell proliferation and modulate apoptosis-related gene expression in MCF-7 and HepG2 human cancer cell lines. Molecules.

[21] Neagu E., Radu G.L., Albu C., Paun G. (2018). Antioxidant activity, acetylcholinesterase and tyrosinase inhibitory potential of Pulmonaria officinalis and Centarium umbellatum extracts. Saudi J. Biol. Sci..

[22] Greggio E., Bergantino E., Carter D., Ahmad R., Costin G.E., Hearing V.J., Clarimon J., Singleton A., Eerola J., Hellström O. (2005). Tyrosinase exacerbates dopamine toxicity but is not genetically associated with Parkinson’s disease. J. Neurochem..

[23] Eruygur N., Koçyiğit U., Taslimi P., Ataş M., Tekin M., Gülçin İ. (2019). Screening the in vitro antioxidant, antimicrobial, anticholinesterase, antidiabetic activities of endemic Achillea cucullata (Asteraceae) ethanol extract. S. Afr. J. Bot..

[24] Borai I.H., Ezz M.K., Rizk M.Z., Aly H.F., El-Sherbiny M., Matloub A.A., Fouad G.I. (2017). Therapeutic impact of grape leaves polyphenols on certain biochemical and neurological markers in AlCl3-induced Alzheimer’s disease. Biomed. Pharmacother..

[25] Rizk M., Borai I., Ezz M., El-Sherbiny M., Aly H., Matloub A., Fouad G. (2018). Possible therapeutic role of grape (Vitis vinifera) leaves polyphenolic extract in the regression of aluminium-induced Alzheimer’s disease in rats. J. Mater. Environ. Sci..

[26] De Castro M.L., Garcıa-Ayuso L. (1998). Soxhlet extraction of solid materials: An outdated technique with a promising innovative future. Anal. Chim. Acta.

[27] Herrero M., Ibáñez E., Señoráns J., Cifuentes A. (2004). Pressurized liquid extracts from *Spirulina platensis* microalga: Determination of their antioxidant activity and preliminary analysis by micellar electrokinetic chromatography. J. Chromatogr. A.

[28] Kaufmann B., Christen P. (2002). Recent extraction techniques for natural products: Microwave-assisted extraction and pressurised solvent extraction. Phytochem. Anal..

[29] Todaro L., Russo D., Cetera P., Milella L. (2017). Effects of thermo-vacuum treatment on secondary metabolite content and antioxidant activity of poplar (*Populus nigra* L.) wood extracts. Ind. Crops Prod..

[30] Lin Y.-S., Chen H.-J., Huang J.-P., Lee P.-C., Tsai C.-R., Hsu T.-F., Huang W.-Y. (2017). Kinetics of tyrosinase inhibitory activity using Vitis vinifera leaf extracts. Biomed. Res. Int..

[31] Betés-Saura C., Andrés-Lacueva C., Lamuela-Raventós R.M. (1996). Phenolics in white free run juices and wines from Penedès by high-performance liquid chromatography: Changes during vinification. J. Agric. Food Chem..

[32] Fernández E.C., Rajchl A., Lachman J., Čížková H., Kvasnička F., Kotíková Z., Milella L., Voldřich M. (2013). Impact of yacon landraces cultivated in the Czech Republic and their ploidy on the short-and long-chain fructooligosaccharides content in tuberous roots. LWT Food Sci. Technol..

[33] Milella L., Caruso M., Galgano F., Favati F., Padula M.C., Martelli G. (2011). Role of the cultivar in choosing *Clementine* fruits with a high level of health-promoting compounds. J. Agric. Food Chem..

[34] Hornedo-Ortega R., Krisa S., García-Parrilla M.C., Richard T. (2016). Effects of gluconic and alcoholic fermentation on anthocyanin composition and antioxidant activity of beverages made from strawberry. LWT Food Sci. Technol..

[35] Brand-Williams W., Cuvelier M.-E., Berset C. (1995). Use of a free radical method to evaluate antioxidant activity. LWT Food Sci. Technol..

[36] Ou B., Hampsch-Woodill M., Prior R.L. (2001). Development and validation of an improved oxygen radical absorbance capacity assay using fluorescein as the fluorescent probe. J. Agric. Food Chem..

[37] Cerezo A.B., Cuevas E., Winterhalter P., Garcia-Parrilla M., Troncoso A. (2010). Isolation, identification, and antioxidant activity of anthocyanin compounds in *Camarosa strawberry*. Food Chem..

[38] Russo D., Valentão P., Andrade P., Fernandez E., Milella L. (2015). Evaluation of antioxidant, antidiabetic and anticholinesterase activities of *Smallanthus sonchifolius* landraces and correlation with their phytochemical profiles. Int. J. Mol. Sci..

[39] Milella L., Milazzo S., De Leo M., Vera Saltos M.B., Faraone I., Tuccinardi T., Lapillo M., De Tommasi N., Braca A. (2016). *α*-Glucosidase and *α*-amylase inhibitors from *Arcytophyllum thymifolium*. J. Nat. Prod..

[40] Di Petrillo A., González-Paramás A.M., Era B., Medda R., Pintus F., Santos-Buelga C., Fais A. (2016). Tyrosinase inhibition and antioxidant properties of Asphodelus microcarpus extracts. BMC Complementary Altern. Med..

[41] Saltos M.B.V., Puente B.F.N., Faraone I., Milella L., De Tommasi N., Braca A. (2015). Inhibitors of *α*-amylase and *α*-glucosidase from *Andromachia igniaria* Humb. & Bonpl. Phytochem. Lett..

[42] Sun T., Tanumihardjo S. (2007). An integrated approach to evaluate food antioxidant capacity. J. Food Sci..

[43] Silla E., Arnau A., Tunon I. (2001). Solvent Effects on Chemical Systems.

[44] Do Q.D., Angkawijaya A.E., Tran-Nguyen P.L., Huynh L.H., Soetaredjo F.E., Ismadji S., Ju Y.-H. (2014). Effect of extraction solvent on total phenol content, total flavonoid content, and antioxidant activity of *Limnophila aromatica*. J. Food Drug Anal..

[45] Matloub A.A. (2018). Optimization of polyphenol extraction from Vitis vinifera L. leaves, antioxidant activity and its correlation with amelioration effect on AlCl3-induced Alzheimer’s disease. Arch. Pharm. Sciences Ain Shams Univ..

[46] Wang L., Weller C.L. (2006). Recent advances in extraction of nutraceuticals from plants. Trends Food Sci. Technol..

[47] Aouey B., Samet A.M., Fetoui H., Simmonds M.S., Bouaziz M. (2016). Anti-oxidant, anti-inflammatory, analgesic and antipyretic activities of grapevine leaf extract (*Vitis vinifera*) in mice and identification of its active constituents by LC–MS/MS analyses. Biomed. Pharmacother..

[48] Aguilar T., Loyola C., de Bruijn J., Bustamante L., Vergara C., von Baer D., Mardones C., Serra I. (2016). Effect of thermomaceration and enzymatic maceration on phenolic compounds of grape must enriched by grape pomace, vine leaves and canes. Eur. Food Res. Technol..

[49] Amarowicz R., Narolewska O., Karamac M., Kosinska A., Weidner S. (2008). Grapevine leaves as a source of natural antioxidants. Pol. J. Food Nutr. Sci..

[50] Guidoni S., Mannini F., Ferrandino A., Argamante N., Di Stefano R. (1997). The effect of grapevine leafroll and rugose wood sanitation on agronomic performance and berry and leaf phenolic content of a Nebbiolo clone (*Vitis vinifera* L.). Am. J. Enol. Vitic..

[51] Monagas M., Hernández-Ledesma B., Gómez-Cordovés C., Bartolomé B. (2006). Commercial dietary ingredients from *Vitis vinifera* L. leaves and grape skins: Antioxidant and chemical characterization. J. Agric. Food Chem..

[52] Weber B. (1992). Phenolische komponenten des weinrebenblattes: Identität und phytopathologische Bedeutung. Ph.D. Thesis.

[53] Uysal S., Zengin G., Aktumsek A., Karatas S. (2016). Chemical and biological approaches on nine fruit tree leaves collected from the Mediterranean region of Turkey. J. Funct. Foods.

[54] Dekdouk N., Malafronte N., Russo D., Faraone I., De Tommasi N., Ameddah S., Severino L., Milella L. (2015). Phenolic compounds from *Olea europaea* L. possess antioxidant activity and inhibit carbohydrate metabolizing enzymes in vitro. Evid. Based Complement. Alternat. Med..

[55] Chew Y.L., Lim Y.Y., Omar M., Khoo K.S. (2008). Antioxidant activity of three edible seaweeds from two areas in South East Asia. LWT Food Sci. Technol..

[56] Giacobini E. (2004). Cholinesterase inhibitors: New roles and therapeutic alternatives. Pharmacol. Res..

[57] Orhan I.E., Akkol E.K., Suntar I., Yesilada E. (2019). Assessment of anticholinesterase and antioxidant properties of the extracts and (+)-catechin obtained from Arceuthobium oxycedri (DC) M. Bieb (dwarf mistletoe). S. Afr. J. Bot..

[58] Kim Y.-J., Uyama H. (2005). Tyrosinase inhibitors from natural and synthetic sources: Structure, inhibition mechanism and perspective for the future. Cell. Mol. Life Sci. CMLS.

[59] Garcia-Jimenez A., Teruel-Puche J.A., Garcia-Ruiz P.A., Saura-Sanmartin A., Berna J., Rodríguez-López J.N., Garcia-Canovas F. (2018). Action of tyrosinase on caffeic acid and its n-nonyl ester. Catalysis and suicide inactivation. Int. J. Biol. Macromol..

[60] Honisch C., Osto A., de Matos A.D., Vincenzi S., Ruzza P. (2020). Isolation of a tyrosinase inhibitor from unripe grapes juice: A spectrophotometric study. Food Chem..

